# Unlocking the efficiency of genomics laboratories with robotic liquid-handling

**DOI:** 10.1186/s12864-020-07137-1

**Published:** 2020-10-20

**Authors:** Houriiyah Tegally, James Emmanuel San, Jennifer Giandhari, Tulio de Oliveira

**Affiliations:** 1 Kwazulu-Natal Research and Innovation Sequencing Platform (KRISP), College of Health Sciences, K-RITH Tower Building, Nelson R Mandela School of Medicine, University of KwaZulu-Natal, 719 Umbilo Road, Durban, South Africa; 2 Department of Global Health, University of Washington, 908 Jefferson Street, 13th Floor, Seattle, WA 98104 USA

**Keywords:** Automated liquid handling, Workstations, Genomics, Lab automation, Liquid handling systems, Liquid handling robots, Automating life sciences

## Abstract

In research and clinical genomics laboratories today, sample preparation is the bottleneck of experiments, particularly when it comes to high-throughput next generation sequencing (NGS). More genomics laboratories are now considering liquid-handling automation to make the sequencing workflow more efficient and cost effective. The question remains as to its suitability and return on investment. A number of points need to be carefully considered before introducing robots into biological laboratories. Here, we describe the state-of-the-art technology of both sophisticated and do-it-yourself (DIY) robotic liquid-handlers and provide a practical review of the motivation, implications and requirements of laboratory automation for genome sequencing experiments.

## Background

Since the completion of the first human genome in 2003 [1], the scope of genomics science and medicine has really diversified [2]. After the emergence of next-generation sequencing (NGS) sequencing technologies, the costs of DNA sequencing considerably decreased, making it much more accessible to scientists worldwide [3, 4]. Indeed, by 2012, 1000 human genomes were completely sequenced [5] and by 2020 this number rose to over 1 million [6]. This cohort included participants from all over the world and revealed important genomic variants which informed crucial opportunities for research and precision medicine [6]. Today, whole genome and whole exome sequencing (WGS, WES) are becoming routine practices in academic, medical and industrial laboratories [7].

Despite an overall drop of costs associated with the sequencing technologies exceeding expectations of Moore’s law [4], there are still major hurdles in the human-led stages of this process. Sample preparation steps in laboratories can be quite time-consuming, tedious and repetitive and are often considered the bottleneck of DNA sequencing [8]. A study of the applicability of genomic analysis to routine cancer diagnosis in the UK revealed that all-manual laboratory processing for NGS results in a turnaround time of as much as 6 days from a request for molecular diagnostics to a genomics report [9]. This is quite long, considering that manual processing would potentially allow operations to only be scaled up to a dozen samples at once. While some parts of the workflow, like nucleic acid extraction, has already been automated [10], preparing the reagents and plates for the extraction still mostly relies on manual labor. In addition to being repetitive and error-prone, this translates to important time and cost inefficiencies for genomics laboratories [11]. These might explain why many laboratories are still finding it difficult to reach the promised $1000 genome [12].

**Fig. 1:**
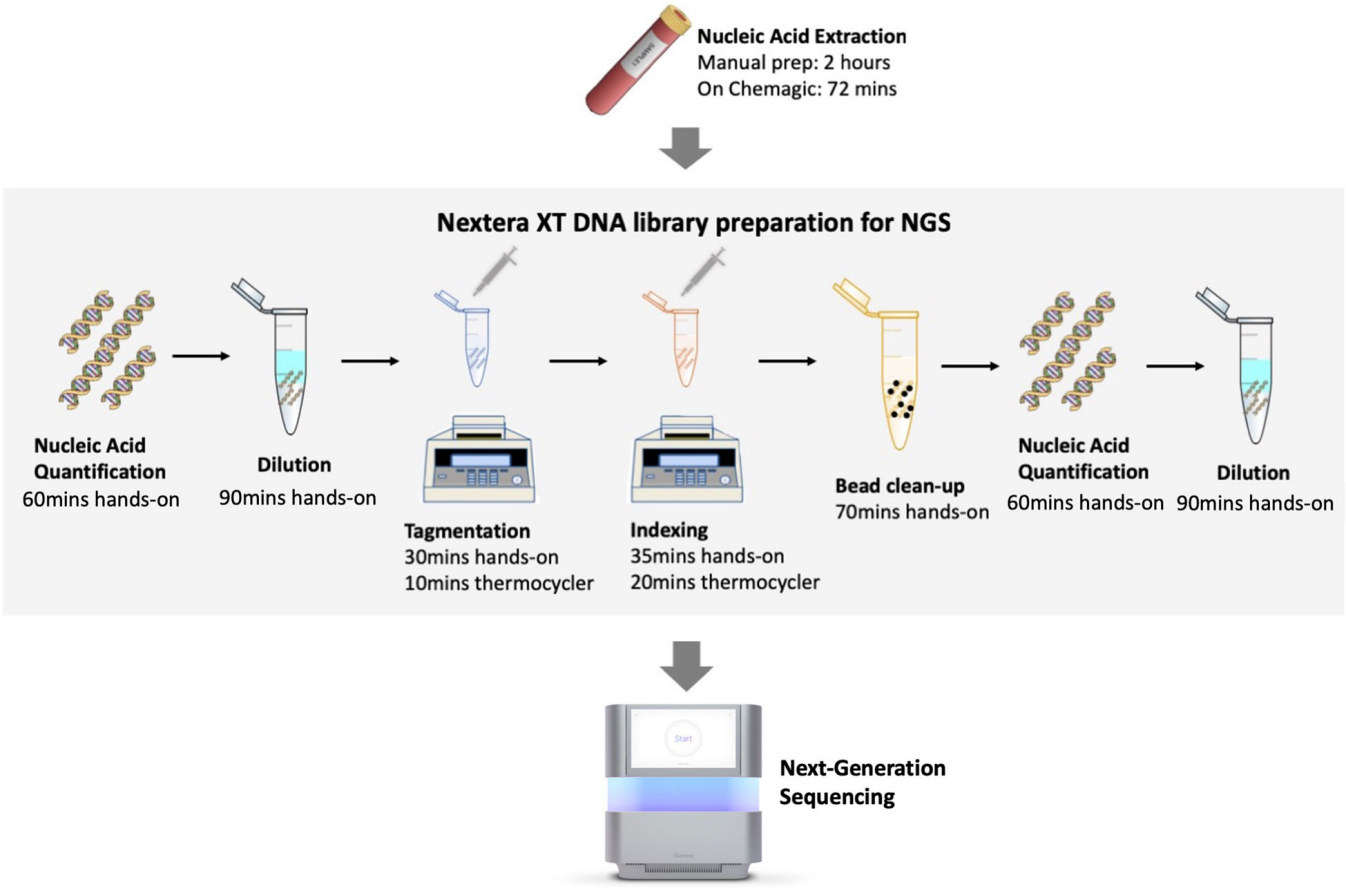
Time Frame for Next Generation Sequencing Workflow for cancer genomics. Showing an estimate of time taken to get 96 samples ready for Next-Generation Sequencing (NGS). In this protocol, automated nucleic acid extraction (on Chemagic) is already shown to speed up the first step. The goal of complete liquid handling automation, considered in this review, would be to significantly reduce the hands-on time for the library preparation steps, before transferring samples to a sequencer. (All images depicted in this figure are our own or adapted from images with no copyright)

In an attempt to further streamline and reduce costs of sequencing, laboratory automation could be the solution. Automation of liquid handling in particular, will improve the performance of high-throughput laboratories in a cost-effective manner [13–16]. In fact, an analysis of the cost breakdown for genome sequencing reveals that 15% of that total cost relates to laboratory personnel in a conventional clinical laboratory [17]. When the same type of cost analysis was carried out for laboratory settings having a Hamilton Microlab STARlet for automated sample preparation, salaries for laboratory staff dropped to only 4% of the total cost [12]. Beyond cost, robots can also carry out tedious and repetitive tasks tirelessly and accurately, presenting a huge advantage over manual liquid-handling. Not only would this help cut down costs associated with manual labor, it would also mean that highly skilled life scientists would not have to spend long hours pipetting liquids anymore. Figure 1 shows an estimate of the hands-on time requirements of the Nextera workflow for sequencing of 96 samples. The library preparation steps alone involve almost 8 h of hands-on operations (Fig. 1). On benchmarking, automation with the Agilent Bravo NGS workstation, for instance, was found to cut down hands-on time on NGS library preparation all the way from 375 to only 25 min [18]. PerkinElmer for its part reports the construction 96 libraries in 3 h and 40 mins with just 10mins of hands-on time [19]. Clearly, automation of library preparations alone could save valuable time for scientists, who could invest this in more productive and intellectually stimulating tasks. Furthermore, the results would be consistent and of a higher quality.

Laboratory automation is not new to life sciences. For years, large-scale pharmaceutical companies have used liquid-handling robots for high-throughput drug discovery and developments [20–23]. Robots have allowed millions of compounds to be screened in short amounts of time for the identification of a single candidate drug. Robots can dispense small and precise volumes of highly fragile and precious bioactive samples. Assays of the pharmaceutical industry can also be easily programmed for automation as these are set protocols that need to be applied repetitively. Industrial-size genomics laboratories have also adopted automation to boost productivity [24]. For instance, to sift through the large number of protein coding genes for protein structure determination, a number of experiments with varying conditions are required [25]. The integration of microarray experiments and automation has also enabled high-throughput analysis of sequences or simultaneous monitoring of thousands of genes in large biotech companies [25].

This review provides an in-depth discussion of robotic liquid-handling technologies and their relevant modalities in the context of the needs of DNA sequencing laboratories. An understanding of this robotic field and its application towards genomics sciences will hopefully help in prompting automation in life science laboratories, with the overarching goal of unlocking the efficiency of genome sequencing.

## Main text

### Overview of technologies

Laboratory automation technologies have been developed for every stage of the laboratory workflow, mainly to suit the needs of big industrial companies. In this review, we focus primarily on the usefulness of liquid-handling systems in genomics research to reduce costs and improve efficiency of sequencing, considering how essential liquid filling, dispensing, mixing and transferring are to genomics research. DNA samples, primers and reagents usually have to be distributed into wells, mixed with substrates or diluted in preparation for amplification and sequencing [26]. There are many different library preparation methods available depending on the application. It includes preparation of libraries where DNA or RNA molecules are ligated with adapters for sequencing [27]. Library preparation methods can vary from those requiring fragmentation (either enzymatic or mechanical), to those requiring A-tailing and adapter ligation or those directly sequenced from cDNA. These steps together with the bead clean-up step, are often the bottleneck in next-generation sequencing (NGS) applications [28] (Fig. 1). A number of state-of-the-art technologies have been developed to achieve accurate liquid dispensing.

**Fig. 2:**
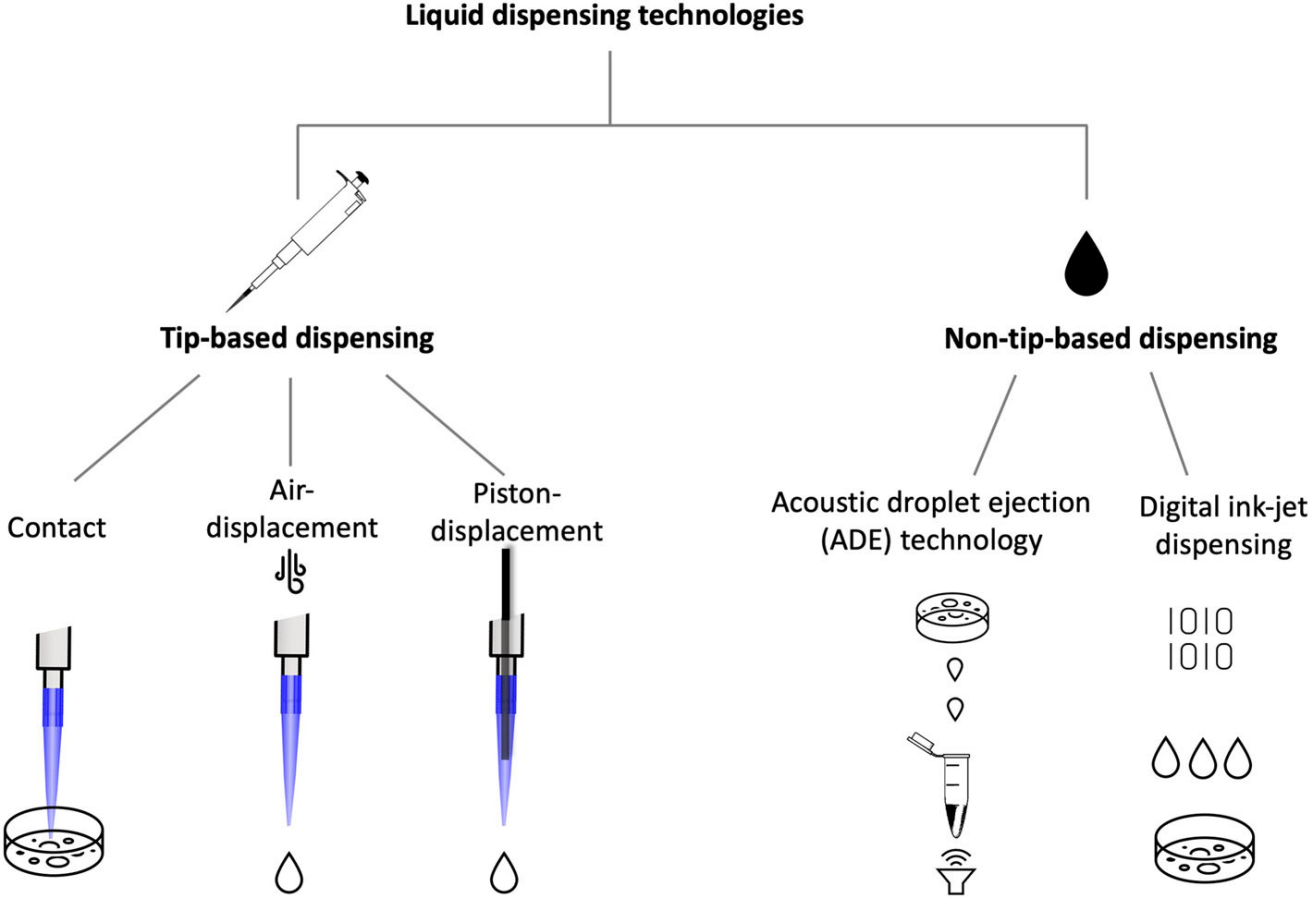
Liquid dispensing technologies divided into tip-based and non-tip-based dispensing. Tip-based dispensing is further classified into contact dispensing, air or piston displacement dispensing, while non-tip-based technologies into acoustic droplet ejection (ADE) and digital inkjet dispensing. (All images depicted in this figure are our own or adapted from images with no copyright)

As the fundamental principle, dispensing has to overcome surface adhesion and, for small volumes relevant to genomics experiments, gravity alone cannot do that [29]. Automated dispensing technologies use several methods, classified into tip-based and non-tip-based dispensing technologies (Fig. 2), all with their own advantages and disadvantages. Tip-based dispensing is the most common, usually requiring a plastic tip from which the liquid is ejected. One way to dispense the liquid from a tip is through a type of contact dispensing, requiring only a touch-off of the dispending tip to detach the liquid. This method is considered to be reliable, simple and low-cost but runs the danger of damaging the tip and pipette from hard contact [30]. Most pipettes on the market currently employ the air-displacement method [31], which uses an air-cushioning to move liquid through the tip. While it does not require contact, this method can produce inconsistent results depending on tip-manufacturing, and can cause microbubbles in the destination solution, hence compromising on dispensing precision and accuracy. Some systems also use a sliding piston to achieve liquid displacement. This is known to be far more accurate than air-displacement systems, especially for high-density or high-vapor pressure liquids, but is often a more expensive set-up.

Alternatives to tip-based dispensing also exist. Acoustic droplet ejection (ADE) technology involves completely contactless liquid handling using sound energy [32]. To eject a small volume droplet from a well, acoustic energy is focused near the surface of the liquid where the frequency of the acoustic wave determines the volume of the droplet. These types of tipless transfers possess the major benefit of eliminating cross-contamination issues. Compared to tip-based dispending, acoustic liquid handling has also been shown to lead to more accurate biological activity in pharmacological assays [33]. This might be due to possible interactions between additives in the plastic tips and reagents, which have been observed to possibly leach from laboratory plasticware [33]. This type of dispensing is also quite gentle, which allows the transfer of delicate proteins, DNA and live cells without loss of integrity. Finally, digital dispensing, using inkjet printing technology, allows the distribution of independently dosed droplets into individual wells, without the need for serial dilution. This provides major flexibility and dispensing precision at very low volumes.

**Fig. 3:**
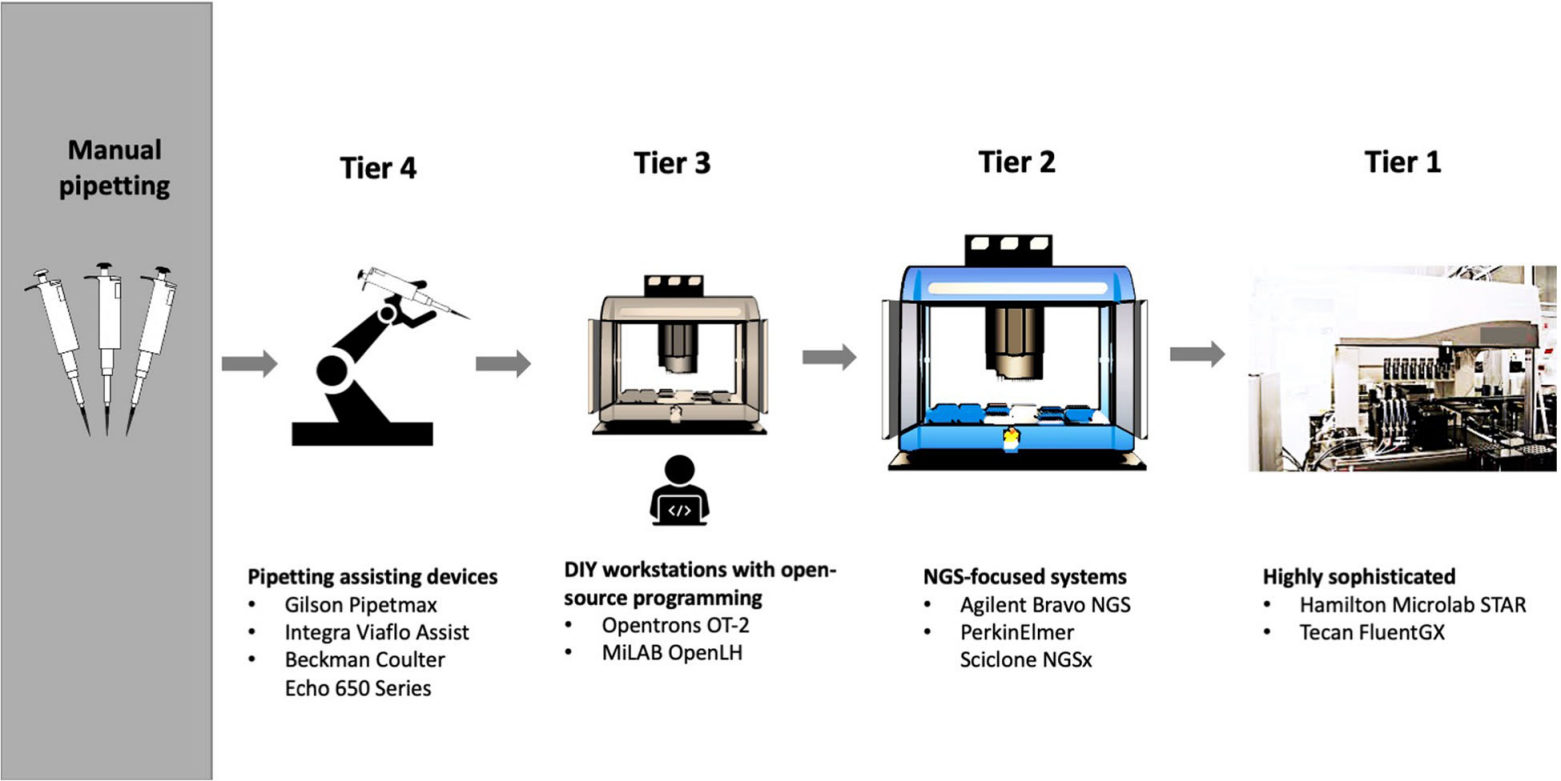
Different categories of liquid-handling robots. Automated liquid-handling systems range from highly sophisticated, such as Tecan FluentGX and Hamilton microlab STAR (Tier 1). To focused NGS sequencing systems, such as the Agilent Bravo and the PerkinElmer Sciclone NGSx iQ (Tier 2). Automated liquid handling can also come in the form of DIY workstations with open-source programming like the Opentrons OT-2 (Tier 3), or simply as pipetting assisting devices such as Gilson Pipetmax and Hudson SOLO (Tier 4), which serves to decrease manual pipetting without fully automating the s. Tier 3 and Tier 4 are often low-cost investments compared to Tier 1 and Tier 2. (All images depicted in this figure are our own or adapted from images with no copyright)

Robotic liquid-handling devices include both handheld devices and workstations. Automated syringes and pipettes have been common practice in life science for some time [25] partly tackling the repetitiveness of sample preparations. However, these do not completely eliminate human involvement, therefore only marginally reducing error and making the experiments less tedious. Robotic systems, on the other hand, can be completely independent once the experiment is running. They can also work tirelessly and consistently without compromising on performance and accuracy, provided that calibration is correct. Robotic liquid-handling workstations come in various scales and set-ups (Fig. 3). They consist of a number of components integrated together into a specific system architecture [30]. All robots must have a control center (to govern its movements), a dispensing head, the mechanical engines, actuators (to control liquid flow) and a substrate deck. Some robots will have sensors installed to monitor the dispensing process and provide feedback control [34, 35]. Robot mechanics work such that they move along x-y axes, and sometimes also along a z drive.

### Modalities of liquid-handling systems

Automated liquid-handling systems have to fulfill some general requirements such as high throughput, high accuracy and precision, especially with low volumes in order for it to be used in life science laboratories. In addition, such robots on the market possess a variety of other properties which make them more or less suitable to certain needs in the laboratory. Therefore, when identifying the most suitable automated liquid-handling workstation, a number of modalities have to be considered and weighed against the specific requirements of a laboratory.

#### Scale of automation

Defining the scale of automation needed should be the first step in selecting an appropriate system. An important distinction to make is whether the need is for single or multi-tasking of liquid handling. Single tasking means that the robot would be used to repetitively undertake one task (e.g. dispensing reagents into multi-well plates) and leaving the rest to humans. This describes a semi-automated approach as opposed to a highly automated approach where multi-tasking is required of the liquid-handling systems. Multi-tasking approaches may incorporate reagent transfer, sample preparation, shaking, experiment result detection and storing. This normally means the integrated automation of single-tasking liquid-handling along with added accessories such as pumps, shakers, plate readers, centrifuges, heating blocks and thermo-cycling. Depending on common sequencing operations, there might even be instances where a laboratory may require two sets of robots, although that would double the initial costs, to distinguish library preparation phases and avoid contamination.

The micro10x Reagent Dispenser from Hudson robotics, as an example, has only one job, which is to fill multi-well plates with reagents quickly (100ul in 96 wells in 10s) and with high precision (coefficient of variation, CV = 0%) [36]. This would be perfect for a laboratory seeking just this level of automation. For workflows requiring higher degrees of automation, a fully-fledged robotic workstation combining liquid-handling with other tasks, such as the Tecan’s Freedom EVO NGS workstation, would be more appropriate [37]. Liquid-handling robots on the market correspond to a whole range of automation needs for NGS. Some, like PerkinElmer’s Sciclone NGSx iQ Workstation, have gone a step further by offering on-deck thermo-cycling and tip-box storage for complete hands-off automation of NGS protocols [38]. Agilent’s Bravo NGS, unlike many other robots, has an advanced microplate managing system, which can include a thermal microplate sealer, a centrifuge and a plate barcode labeler [39].

#### Workflow

The next aspect to consider is typically the workflow for which the liquid-handling robot is required. The liquid volumes, labware formats (tubes,wells etc.), type of tips, and any additional equipment (pumps/shakers) to be used in the relevant protocol(s) are all determinants of what features the robot would need to possess.

#### Flexibility

Liquid-handling workstations available on the market allow for different degrees of flexibility and modularity (Table 1). The need for flexibility in workflows is a crucial consideration in choosing a robot. For a laboratory that routinely runs different protocols, the question of how easy it would be to incorporate other workflows to the liquid-handler’s operations should be a top priority. Some manufacturers, such as Hamilton Robotics, allow for the autonomous programming [48] of the robot’s workflow while others, like Tecan provide a set of pre-programmed protocols and will require the consultation of one of their engineers to incorporate new ones to the workstation [40]. Open source programming robots boast at being the most flexible liquid-handling systems. Opentrons robots are fully modular in terms of protocol design and new protocols or labware can be easily coded into a versatile programmatic interface [49]. Flexibility should also be considered in terms of the consumables that can be used. On some robots, generic tips, plates and tube holders can be fitted while on others, only equipment from the same manufacturer can be used, which could be a limitation on the long run. Hamilton robots, for example, must imperatively use Hamilton consumables designed to give the best performance and accuracy within the workstation [41]. However, this can make the process more expensive as one is locked with a sole supplier. There is also the question of modularity of the workstation, that is, whether it would be necessary to extend or modify the workstation hardware components (e.g. pumps, washers, different pipetting arms) and how straightforward that would be to achieve. PerkinElmer’s Sciclone NGSx iQ Workstation, for instance, can even be fitted with on-deck thermal cycling, which extends the hands-off experience further to clean-up PCR steps [38].

**Table 1:** Summary of features on some important liquid-handling workstations or pipetting assisting devices available on the market. Pipetting precision is measured in coefficients of variation (CV) and accuracy is measured as a percentage (R) [18, 29, 38, 40–47]. *Quotations were obtained in July–August 2020 from suppliers in South Africa and prices converted to USD at the prevalent exchange rates

Company	Workstation	Size (cm)	Pipetting set-up	Dispenced volumes(μl)	Pipetting precision (CV)	Pipetting Accuracy (R)	Control Centers	Programming	Dispensing speed (Pipetting Assisting Devices)	Time required to complete 96 libraries (Workstations)	Consumables	Monitoring of dispensed volumes	Number of deck positions	Decontamination options	Blockage detection and log file generation	Price (USD)*
Hamilton Robotics	Mircrolab STAR	166.4 × 79.5 × 90.3	8 independent channels of dynamic volume range	1–1000	6–1.5%	10–2%	INSTINCT+VENUS	User-programmable: graphical interface to create methods	ND	8 h hands-off	Hamilton consumables	liquid level detective using conductive or pressure-based methods	45	HEPA filter hoods with UV light + washing stations	pressure or capacitance-based clot detection	123,605
	Microlab NIMBUS96	104.6 × 70.9 × 83.1	96–Channel Multi-Probe Head of dynamic volume range	1–1000	5–1%	5–1%	NIMBUS software	User-programmable: graphical interface to create methods	ND	ND	Hamilton consumables	liquid level detective using conductive or pressure-based methods	up to 20	HEPA filter hoods with UV light + washing stations	can detect clots and offers real-time tracking of the aspiration performance	ND
Beckman Coulter	Biomek i5	112 × 81 × 112	0.5 μL-1000 μL (Multichannel) or 0.5 μL-5000 μL (Span-8) pipetting volume ranges - Single Multichannel head (96/384) or Span-8 pipetting with gripper	5–1000	< 5.6%	< 2.83%	DART 2.0	Both manufacturer and user programmable	ND	ND	specific Biomek pipette tips	Conductive tips enable liquid-level sensing.	25	Not autoclavable	Cameras enable live broadcast and on-error video capture	128,530
Beckman Coulter	Biomek 4000	122.5 × 50.5 × 67.5	single/multi channel options	1–1000	< 5.6%	< 2.83%	DART 2.0	Both manufacturer and user programmable	ND	ND	specific Biomek pipette tips	ND	ND	ND	ND	ND
Tecan	Fluent GX (480/780/1080)	123.6 × 115 × 785 / 123.6 × 165 × 785 / 123.6 × 215 × 785 (depending on model)	8 independent channels of various volume ranges	0.5–1000	8–0.3%	10–0.5%	ND	ND	ND	ND	Tecan-specific	Liquid level detection	30/48/72 (depending on model)	An integrated laminar flow HEPA hood with UVC light maintains a clean environment throughout the workdeck	User interaction logs	ND
Eppendorf	epMotion 5075vt	107 × 61 × 67	4 exchangable dispensing tool (single/multi channel)	0.2–1000	3–0.15%	0.35%	epBlue	ND	ND	6.2 h	Ependorf consumables, which are pretty generic	optical sensor	15	CleanCap option for UV decontamination and HEPA air filter	debug and log files	170,078
Perkin Elmer	Sciclone NGSx/IQ workstation	170.8 × 90.7	96-channel	1–200	< 5% (1-5ul), < 2%(5-200ul)	ND	Sciclone Maestro software	Both manufacturer and user programmable	ND	8 h	Sciclone-specific	ND	24	ND	Error handling and recovery in an intuitive format	225,082
Agilent Technologies	Bravo NGS	112.1 × 55 × 188	96/384 Channel Disposable Tip Head	0.3–250	5%	± 10%	Agilent Vworks		ND	8 h (only 25 mins hand-on)	Agilent-specific	ND	9	ND	ND	165,000
Thermo Fisher Scientific	Multidrop Combi Reagent Dispenser	25.5 × 33 × 22	syringe+ 8 channel cassette 0.5-50 μL/8 channel cassette 5-2500 μL	0.5–2500	10–1%	2 μl: ± 10%, 10 μl: ± 5%, > 10 μl: ± 5%, 20 μl: ± 2%, > 100 μl: ± 1%	Serial RS-232C (USB)	ND	96-well plate: 10 μL in 3 s.; 20 μL in 4 s.; 100 μL in 10 s |. 384-well plate: 1 μL in 5 s.; 5 μL in 5 s.; 10 μL in 6 s.; 20 μL in 9 s | 1536-well plate: 1 μL in 14 s.; 5 μL in 26 s.	ND	plastic tube tips	ND	ND	cassettes are autoclavable	ND	18,437
Gilson	Pipetmax	54.4 × 65.5 × 53.1	8 channel 1 μL - 20 μL, 8 channel 20 μL - 200 μL, Single channel 1000 μL	1–1000	8–1%	5–0.25%	TRILUTION	User-programmable	ND	4hrs18mins	generic	ND	ND		ND	47,761
Hudson Robotics	SOLO	74.9 × 49.5 × 61	Single channel: 1uL to 10 mL; optional pump assembly for bulk dispensing - 8 & 12 channel: 50 uL, 100 uL, 200 uL, 300 uL and 1000 uL pipette heads	0.2–10,000	2uL: < 2%, Larger volumes: < 0.5%.	ND	Solosoft Control	User-programmable	30mins/copy 96-well plate, with tip changes every time	ND	generic	Liquid level detection optional	4 to 12	Microplate washer optional	ND	
Opentrons	OT-2	63 × 57 × 66	2 interchangable channels	0.5–1000	ND	ND	Opentrons Python API	User-programmable	Fills a 96-well plate in 20 s	ND	Opentrons-specific or generic	Not available	9	ND	ND	5000
miLAB (The Media Innovation Lab, University of Hezliya, Israel)	OpenLH	ND	1 channel of choice	0.5–50	ND	ND	Blockly Python API	User-programmable	ND	ND	generic	Not available	ND	ND	ND	1000
Integra ViaFlow Assist	Integra Biosciences	34 × 36 × 40	single (0.5-5000ul) or multi-channel (0.5-1250ul)	ND	≤ 1.0%	± 1.5%	VIALINK	Both manufacturer and user programmable	ND	ND	generic	artificially tracks liquid level by using tip travel distances	4	Manually. Components can be autoclaved	ND	6750
Beckman Coulter	Echo 650 Series	53.9 × 68.3 × 92.5	Echo acoustic droplet ejection (ADE) technology	0.0025–5	< 8%	< 10%	ND	ND	ND	ND	ND	liquid level detection by using low energy sound waves	ND	ND	ND	ND

#### Pipetting channels

The pipetting set-up can vary considerably among the liquid-handling workstations. A laboratory looking to transition to automation needs to carefully consider which pipetting ranges and the number of channels that would be suitable. Liquid-handling workstations can typically accommodate a certain arrangement of two single or multi-channel pipetting arms of specific volume ranges at a time. These are usually interchangeable for pipettes of other volumes. Some more sophisticated models, such as the Hamilton Microlab NIMBUS96, can have up to eight independent channels with a dynamic pipetting range from 0.5ul to 1000ul [50]. Agilent’s Bravo NGS, for its part, comes with interchangeable 96 or 384 channel pipetting heads, which translates to an incredible versatility of operations, especially focused to NGS library preparations in microplates of corresponding sizes [18].

#### Control centers

The robot operations are usually coordinated from control centers. Whether the robot can accommodate new protocols or have fixed operations, it is essential to consider the system’s usability. In life science laboratories, where engineers or programmers are somewhat scarce, the sustainability of automated liquid-handling systems will rely on how user-friendly they are for daily use. Most of them can be managed from software installed to an attached tablet and can be operated from the touch screens. Older or less sophisticated model might still require a connected computer, which could make the whole set-up bulkier. In cases where the workstation operations can be customized by the user depending on evolving protocol needs, the process of programming the workflow also have to be as straightforward as possible. Most newer models are proposing control centers where workflow can be designed or modified using drag and drop icon-based tasks. The Hamilton Microlab STAR and the Hudson SOLO robots both use this graphical approach to make their systems approachable to biologists [44, 48]. Opentrons robots can even be controlled using a fully programmatic python API, which although not as accessible as graphical protocol designers, can be a real asset towards customizing [51]. Often, these control centers will also keep a detailed record of stepwise operations carried out by the system, which can be used for any error-handling.

#### Washing and decontamination

In genomics laboratories, risk of contamination is one of the biggest concerns [52], which should be minimized to the best of abilities. Liquid-handling robots typically come with washing modules to perform cleaning up of the robot head after use [53]. Depending on the vendor, there might also be the option of adding a microplate washer for well plates. The washing modules usually consist of pumps passing water or detergents through the robot head or labware and aspiration of waste. For workstations without washing systems, it is usually recommended to run a washing solution through the pipettes as a way to clean. Another option is to consider pipetting robots which make use to disposable tips and labware, hence reducing the need for cleaning. Yet, some experiments, for example involving microbial samples, might require thorough decontamination [54]. In such cases, higher-end workstations might be preferable as they usually come with an integrated UV light module. In the case of acoustic droplet ejection systems, such as the Echo 650 series, liquid dispensing is contact-less which largely eliminates any source of contamination [32]. There is also the distinction between open and enclosed systems, given that open systems, those not enclosed within four walls, would definitely be more susceptible to contaminations from the environment.

#### Precision & accuracy

In life sciences, and in genomics particularly, high degrees of precision and accuracy in pipetting volumes are required to guarantee the success and reliability of experiments. Precision refers to the consistency of the pipetting equipment while accuracy refers to the trueness of the volume handled. Pipetting errors might lead to misleading measurements of DNA concentrations, for instance. It becomes even more tricky as, very often, genomics protocols require small volumes of highly precious samples and reagents, which allows for virtually no margin of error. For this reason, liquid-handling workstations are normally fitted with various technologies to achieve precise pipetting. Hamilton’s robots, for example, uses a patented lock-and-key method to tightly seal the pipetted and tips together to ensure accuracy [41]. The Hamilton Microlab STAR can also detect liquid levels dispensed either using conductive tips or pressure-based methods [48]. Other systems, such as in Tecan and Eppendorf workstations, have integrated sensors for contact-free liquid level detection for ensuring precision [40]. The degrees of precision for some of the more sophisticated machines typically range between coefficients of variation (CV) of 2–8% for volumes of the order or 1ul and 0.15–1.5% for larger volumes. A lower coefficient of variance means higher reproducibility. The accuracy of pipetting oscillates between 0.35–10% (regression). The Agilent Bravo NGS, for example, has been reported to produce a CV of pipetting of 11%, compared with 18% for manual preparations [18].

#### Size

Liquid-handling workstations now come in all sizes and formats. Prior to the acquisition of a robot, different options should be considered in relation to their respective dimensions and the available bench space in the laboratory. At times, the portability of the workstations might also be of concern depending on the set-up of the laboratory. More compact robots could, for instance, be desirable in setting up mobile genomics laboratories as a strategy for fast response to infectious disease outbreaks [55]. Conversely, for commercial genomics laboratories, it might be necessary to acquire a workstation with a maximum throughput, which usually means bigger deck sizes able to handle a larger capacity of operations.

#### Throughput

Liquid-handling systems have been designed to deliver a range of throughputs. This is most directly achieved through varying dispensing speeds, the number of channels that can be accommodated on deck, and the size of the deck. Hudson Robotics’ SOLO system, for instance, would take close to half an hour to fill a 96-well plate with tip changes for each well, whereas Hamilton’s Microlab STAR would complete the same task in around 5 min [44, 48]. Speaking of overall turnout, the PerkinElmer Sciclone NGS x iQ and Agilent Bravo workstations both average at a capacity of 96 library preparations per day [18, 38]. Similarly, a workstation such as in the Hamilton range, with eight independent channels of dynamic volume will inevitably perform dispensing of the required volumes faster that a liquid handler than can accommodate only two channels of small volume ranges. Depending on the needs, however, having a system with a single 96 or 384-channel tip head, like the Agilent Bravo NGS, could boost throughput if simple operations like repeat dispensing is required. The different automated workstations also come with varied deck sizes, which can also highly determine overall throughput. The number of deck positions varying from 9 (Opentrons OT-2) all the way up to 72 (Tecan Fluent GX 1080). Some machines even provide on-deck plate or tip-box storage facilities to really optimize walk-away operations (Tecan Fluent GX 1080). However, faster speed of operations, especially if done without compromising on accuracy, will undoubtedly translate to a higher price tag as well. Therefore, a careful consideration for throughput requirements should be made when shifting to laboratory automation. Larger life-sciences companies, for whom high throughput operations are more crucial, might be comfortable in investing in higher quality systems, compared to smaller research laboratories, for whom even low-throughput liquid handling might still be a welcomed upgrade to laboratory activities.

#### Durability

An important consideration for automatic liquid-handling systems is the durability of the machines, given the considerable investment that is the acquisition of one of these. They have to be evaluated as per the ability of their respective mechanics to resist wear and tear. Some of the more sophisticated manufacturers have integrated particular technologies to increase the lifespan of their systems. Hamilton’s tip loading technology, which involves fitting the pipette and the tip seamlessly using a lock and key approach, means that no vertical force is needed to secure the tips [41]. This in turn translates to much fewer mechanical issues compared to other systems having a conventional approach to attaching tips. Moreover, given the specificity, sometimes patented of the technologies employed in these liquid-handling systems, durability will also depend on reliability of the manufacturing companies. Indeed, it is crucial that expert technical support will be available long-term from the vendors. Such reliability is often directly proportional to the duration of time the companies have been on the market and the success they have had broadly within the field.

#### Cost

Acquiring a liquid-handling workstation is a significant investment and budget availability is one of the most important factors to be weighed, given that they all tend to be on the expensive side. Typically, the prices of workstations from established manufacturers will vary approximately between $50,000– $250,000. Table 1 provides pricing information of some individual liquid-handling systems discussed in this review. Inevitably, those with more flexibility, accuracy, and generally better features, will come at a higher price, both in terms of initial costs and ongoing servicing charges. However, what the more expensive robots can offer are not necessarily properties desirable to a certain research group. For instance, if it has been determined that automation is only required for one specific operation or protocol, then flexibility can definitely be compromised for a workstation of lower price.

Another important aspect of budget planning is the cost considerations of associated consumables. While the cost of workstations and pipetting instruments are generally one-time investments, maintenance costs aside, that of consumable labware like tubes, pipette tips and plates are running costs. A distinction is to be made between generic plasticware that form part of any biological laboratory’s general budget and system-specific tips, tubes and plates, like the Hamilton’s, which come with a higher price-tag (Table 2).

**Table 2:** Pricing information for an estimated use of tips per system for the NGS preparation of 96 libraries

Company	Tips Description	Number of tips per box	Price of box (USD)**	Number of specific tips needed for 96 samples*	Cost for 96 samples (USD)	Total for 96 samples (USD)
Hamilton robotics	50ul CORE TIPS W FILTER	5760	876.52	1920	292.17	385.54
Hamilton robotics	STD. VOL. CORE TIPS FILTER - 300 μl tips with filters	5760	893.77	576	89.38	
Hamilton robotics	Hamilton Robotics - HIGH VOL. CORE TIPS FILTER - 1000 μl tips with filters	3840	613.09	25	3.99	
Ependorf	50 uL, filter, reload tips	2304	584	288	72.94	170.19
Ependorf	300 uL, filter, reload tips	2304	584	288	72.94	
Ependorf	1000 uL, filter, reload tips	2304	583.51	96	24.31	
Gilson	Generic 200ul tips	960	130.25	1514	205.42	296.60
Gilson	Generic 30ul tips	960	130.25	672	91.18	

*Tip usage was estimated based on experience in the laboratory with the Nextera XT DNA library preparation protocol for NGS and in communication with suppliers of each automated system. **Quotations were obtained in July–August 2020 from suppliers in South Africa and prices converted to USD at the prevalent exchange rates

### Big-deck v/s low-cost robotic liquid handling systems

The automatic liquid-handling sphere has seen three main phases of evolution. First-generation liquid-handling workstations rolled out by prominent companies like Qiagen and ThermoFisher were very much built for facilitating operations of the pharmaceutical industry [20]. These processes required maximum throughput but were relatively straightforward to automate. The workstations were designed to exhibit the highest performance on a few set protocols without the need for much user-based modifications. They were so-called closed systems. When the need for automation reached the genomics industry, the requirements shifted. Not only was protocol design now more intricate, it also had to be flexible. The second generation of robotic liquid systems, like the Hamilton robots, therefore, became more elaborate and open at the same time. They started offering the possibility of user-friendly protocol editing and design, and modular deck layouts.

Both first- and second-generation liquid-handling robots mentioned above are what could be termed big-deck systems. They are supplied by established liquid-handling vendors and come at high costs. The main suppliers of big-deck automated liquid-handling systems include Tecan Group, PerkinElmer, Thermo Fisher, Agilent Technologies, Hamilton Robotics, Eppendorf, QIAGEN, and Beckman Coulter. The big-deck systems can be classified as the general highly sophisticated ones, and the NGS-focused ones (Fig. 3).

Pharmaceutical companies and large DNA sequencing service laboratories with a high cash flow are easily able to invest in state-of-the-art equipment. For smaller academic or clinical laboratories with much lower budgets, the reality is unlikely to be similar. That is where the more recent low-cost robotic liquid-handling systems come in (Fig. 3). These can also be considered simply as pipetting assisting devices, examples being the Hudson SOLO robot and the Integra ViaFlo Integra ASSIST robot, which aim to provide comparable liquid-handling accuracy as workstations from established companies for about half the price and without the need to automate the full process [44]. The SOLO can be easily adapted for unique applications and can be fitted with generic laboratory equipment if extension is required [44]. The Integra ASSIST offers a range of pipetting protocols such as serial dilutions, repeat dispensing and variable dispensing with a range of single or multi-channel pipettes and plate set-ups allowing for liquid ranges from 0.5-1250ul in plates of 6-wells to 384-wells [47].

In addition, recent years have seen an emergence of various DIY and open-source liquid-handling robots (Fig. 3). Opentrons, a start-up company, is one of the main players in opening up access to liquid-handling automation to the masses. The company’s latest robot, the OT-2, only costs $5000, which is at least ten times more affordable than big-deck robotic liquid-handling systems [45]. Moreover, Opentrons robots operate under an open-source model, meaning that their design can be adapted in any way necessary. This, as argued by the company, represents true flexibility, both in terms of protocol programming and deck layout. Protocols can easily be coded in Python programming language using available functions. Consumables and labware do not have to conform to any specific standards as long as they fit the generic-sized placeholders on the deck. One can even imagine making DIY labware using laser cutters and 3D printers to allow for any protocol configurations imaginable. Another example is the OpenLH (Open liquid-handling) robot. The OpenLH uses an open source robotic arm, is easy to assemble from completely open-source instructions and costs only around $1000 [56]. While it performs only the most basic pipetting operations, it claims to do so at gold-standard accuracy. At the periphery of liquid-handling for genomics, there are other DIY robots like the EvoBot and PlasmoTron, used for chemical life research and parasite culture respectively [57, 58]. This shows a real momentum towards developing accessible solutions for laboratory automation in the life sciences and is an indication of an endeavor that will continue growing.

At this stage of their evolution, however, low-cost and DIY liquid-handling systems do come with a few downsides. While they do carry out the core functions of automated liquid-handlers, they are usually not equipped with high-tech modules present on their big-deck counterparts to ensure highest throughput, ease of use, accuracy and durability. Unsurprisingly, the most prominent vendors, who have been in this field for years, often hold patents on the technologies that make their machines so desirable. Such state-of-the-art is therefore unlikely to be replicable by start-up companies or DIY engineers in the very near future. Yet, this is not to say that these do not have a place on the market. For small research and clinical laboratories, a low-cost liquid-handler may be a major asset for genomic sample preparations. It all comes down to a careful consideration of the modalities described above in relation to the needs of a specific laboratory.

### Semi-automation

While the big-deck liquid-handling systems offer fully automated walk-away NGS preparations, sometimes even including on-board PCR, these come with a large increase to cost, which often is a roadblock towards acquiring a robotic liquid-handler. Indeed, not only is the initial cost of fully-automated systems much higher, they also have added components that need extremely expensive maintenance, often require the use of system-specific labware – again a large recurrent cost and pose a risk of loss of costly NGS reagents in the event of a breakdown. Maybe one of the best approaches then would be to devise a semi-automated workflow.

A semi-automated system could be a set-up where the heavy repetitive pipetting into 96-well plates is done by the automated liquid-handler and the user transfers pipetted plates to PCR machine manually, offering a much safer and cost-effective compromise. This is where the low-cost and DIY liquid handlers, or pipetting assisting devices, presented above come in handy. While they would require a slightly higher hands-on time than fully automated systems, they come with much cheaper initial price-tags and maintenance costs and still help overcome manual pipetting in NGS experiments. Semi-automation would thus allow laboratory scientists to reap maximum benefits from the available workstations, while not compromising on accuracy, and without having to dedicate exaggerated budgets or risking precious reagent wastage.

### Future of automated liquid-handling

Liquid-handling automation is already finding its place in various aspects of life sciences. Be it in microbiology [59], synthetic biology [60–62], endocrinology [63], or genetics [58, 64–66], laboratory biologists are increasingly trusting automated liquid handling workstations to streamline their protocols. Genomics laboratories at prominent institutions have also already dipped their feet in liquid-handling automation, be it for gene expression, NGS, or third-generation sequencing for a number of diseases [67–83].

However, to achieve the full potential of efficiency and accuracy of liquid-handling automation for genomics laboratories, especially where resources for the most expensive technologies are limited, it is important to address some remaining concerns. One aspect that still needs improvement is evaporation control, which is particularly relevant to the handling of small volumes in genomics workflows [30]. This ideal of long automated protocols requiring no user intervention is still limited by the uncertainty of low liquid volumes resisting evaporation inside of a workstation. While some attempts have been made to mitigate the effects of evaporation, there is more engineering work required to overcome this obstacle in automated liquid-handling. One technique used has been to designate outer wells of a microplate as a dummy well holding working reagents as it is these wells that suffer more from evaporation [84]. Another solution has involved the use of sensors to monitor environmental conditions to monitor and reduce evaporation [85]. Clearly, these are rather basic techniques and do not really eliminate the problem.

Another important limitation of automated pipetting comes into place when viscous liquids are involved, which happens every so often in genomics workflows. Conventional liquid aspiration and dispensing technologies are not presently adapted to viscous materials [86]. This is because the large majority of workstations today use a non-contact approach for liquid dispensing, which does not provide enough force to overcome the forces of viscous liquids sticking to pipette-tip surfaces [86]. Research into this issue has revealed several ways to better handle viscous liquids including adapting the distance from which dispensing happens, and even the technology used for aspiration [87]. However, these have yet to be implemented into commercially available genomics workstations. If these limitations are a concern, using the workstations as part of a semi-automated workflow would again aim to optimize their use while linking up the steps manually where the automated systems fail to show satisfactory performance.

## Conclusion

Automatic liquid-handling systems have the potential to significantly optimize genome sequencing outputs, both in time and costs. As the needs of biological laboratories become clearer, the properties of these pipetting robots also evolve. While aiming for largely similar functionality, each workstation offers various different focused qualities. With the plethora of machines available on the market, the laboratory’s requirements ultimately become the most important consideration when opting for automation. Often, a compromise will have to be reached between the price of such a workstation and its offerings. In that case, laboratory throughput will dictate the projected returns on investment, which again shows the specificity of liquid-handling automation needs.

## Supplementary information

**Additional file 1.**

## Data Availability

Not Applicable.

## Abbreviations

DIY: do-it-yourself
NGS: next-generation sequencing
WGS: whole genome sequencing
WES: whole exome sequencing
ADE: acoustic droplet ejection
